# Improving Vector Evaluated Particle Swarm Optimisation Using Multiple Nondominated Leaders

**DOI:** 10.1155/2014/364179

**Published:** 2014-04-27

**Authors:** Kian Sheng Lim, Salinda Buyamin, Anita Ahmad, Mohd Ibrahim Shapiai, Faradila Naim, Marizan Mubin, Dong Hwa Kim

**Affiliations:** ^1^Faculty of Electrical Engineering, Universiti Teknologi Malaysia, 81310 Johor Bahru, Malaysia; ^2^Faculty of Electrical & Electronic Engineering, Universiti Malaysia Pahang, 26600 Pekan, Malaysia; ^3^Department of Electrical Engineering, Faculty of Engineering, Universiti Malaya, 50603 Kuala Lumpur, Malaysia; ^4^Department of Instrumentation and Control Engineering, Hanbat National University, Daejeon 305-719, Republic of Korea

## Abstract

The vector evaluated particle swarm optimisation (VEPSO) algorithm was previously improved by incorporating nondominated solutions for solving multiobjective optimisation problems. However, the obtained solutions did not converge close to the Pareto front and also did not distribute evenly over the Pareto front. Therefore, in this study, the concept of multiple nondominated leaders is incorporated to further improve the VEPSO algorithm. Hence, multiple nondominated solutions that are best at a respective objective function are used to guide particles in finding optimal solutions. The improved VEPSO is measured by the number of nondominated solutions found, generational distance, spread, and hypervolume. The results from the conducted experiments show that the proposed VEPSO significantly improved the existing VEPSO algorithms.

## 1. Introduction

Multiobjective optimisation (MOO) problems involve the simultaneous minimisation/maximisation of multiple objective functions, which usually conflict with each other. Due to the conflict between objective functions, a single solution could not satisfy all objective functions. Hence, MOO problem usually results in a set of tradeoffs or nondominated solutions.

The vector evaluated particle swarm optimisation (VEPSO) [1] algorithm has been widely used to solve MOO problems [2–7]. As an example, VEPSO algorithm has been implemented in solving DNA sequence problem by minimising four objective functions, namely, *H*_measure_, similarity, continuity, and hairpin, and two constraints, namely, melting temperature and GC_content_ [7]. Compared to DNA sequence design using binary particle swarm optimization which produces single set of DNA sequences [8], VEPSO is able to generate several sets of good DNA sequences which fulfil the four objective functions and two constraints.

The VEPSO algorithm is adapted from the vector evaluated genetic algorithm (VEGA) [9], in which each swarm optimises one objective function by using the best solution from another swarm as a guidance. However, the VEPSO suffers from performance drawback. Therefore, it is improved by redefining the selection of the guidance from nondominated solution, known as VEPSOnds [10]. Although VEPSOnds has shown better performance than conventional VEPSO, the VEPSOnds suffers from weak performance in terms of lacking solution distributions and convergence to the true Pareto front.

Other than VEPSOnds, there are various MOO algorithms which used nondominated solution to guide particle in finding the optimum solutions for MOO problem. For example, in Multiobjective particle swarm optimisation (MOPSO) algorithm [11, 12], all nondominated solutions are separated into groups according to their location in the objective space. A guiding solution for each particle is then randomly selected from the group containing the fewest solutions. Besides, in nondominated sorting PSO (NSPSO) algorithm [13], which uses the main mechanism of the nondominated sorting fenetic algorithm-II [14], each particle is guided by a nondominated solution that is randomly selected using the niche count and the nearest neighbour density estimator. A nondominated solution is selected based on binary tournament selection for the purpose of guiding the other particles in the optimised MOPSO (OMOPSO) algorithm [15]. Additionally, Abido [16] introduces the use of two nondominated solutions, which are called the local set and the global set. The guide is selected based on the nearest distance in objective space between each particle and each member of the nondominated solution of both sets.

Noticeably, most particle swarm optimisation- (PSO-) based MOO algorithms, including conventional VEPSO and VEPSOnds, only use one solution as the particle guide. In particular, in VEPSOnds, particles from a swarm will be guided by the nondominated solution which has the best fitness at one objective function. Thus, the particles may guide the searching with limited information about the other objective functions during the optimisation process. Therefore, VEPSOnds can be further improved by using more than one nondominated solution as particle guide. In this context, this improved VEPSO algorithm will use the best solution from all swarms as guidance during the optimisation process.

The next section of this paper explains the particle swarm optimisation (PSO), the conventional VEPSO, VEPSOnds algorithm, and the proposed VEPSO algorithms. The following section presents the experimental work and the description of the benchmark test problems and performance measures and the discussion of the results. The final section concludes the proposed technique and discusses few possible future works.

## 2. Multiobjective Optimization

For explanation, consider a minimization problem

(1)  minimize  fitness  function,F→(x→)={fi(x→),i=1,2,…,M}  subject  to={gj(x→)≤0,j=1,2,…,phk(x→)=0,k=1,2,…,q,

where $\vec{x}=\{x_{1},x_{2},\ldots ,x_{n}\}$ is the decision variable vector which represents the possible solution, *M* is the number of objectives, and *f*_*i*_ ∈ *R* ^*n*^ → *R* is the objective function.  {*g*_*j*_, *h*_*k*_} ∈ *R* ^*n*^ → *R* are the inequality and equality constraint function, respectively. The Pareto optimality concept is defined as follows.

**Definition 1**

**Figure 1:**
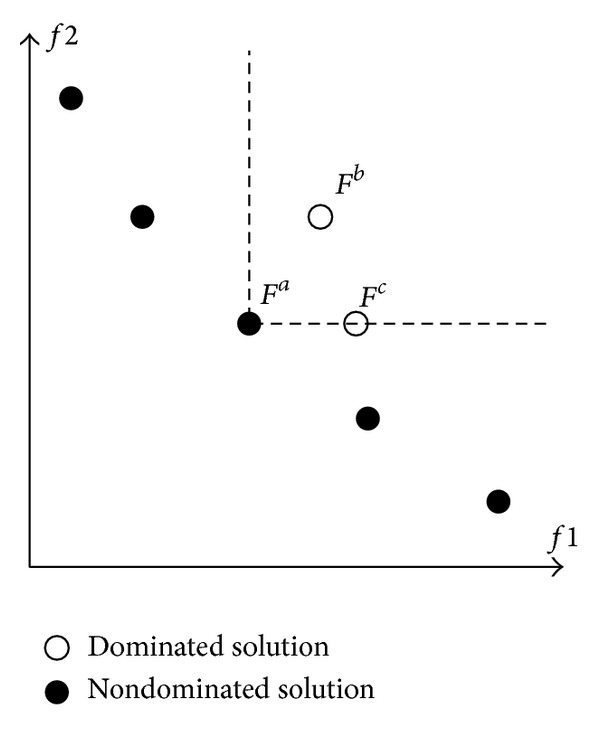
Dominance relation for two-objective problem.

Given $\{\vec{F^{a}},\vec{F^{b}}\}\in R^{m}$ as two vectors, $\vec{F^{a}}$ *dominates* $\vec{F^{b}}$ (denote as $\vec{F^{a}}≺\vec{F^{b}}$) if and only if *f*_*i*_^*a*^ ≤ *f*_*i*_^*b*^ for *i* = 1,2,…, *m* and *f*_*i*_^*a*^ < *f*_*i*_^*b*^ for at least once. Dominance relation of $\vec{F^{a}}≺\vec{F^{b}}$ and $\vec{F^{a}}≺\vec{F^{c}}$ can be illustrated as the labelled circles in Figure 1 for a two-objective problem.

**Definition 2**

A decision variable vector $\vec{x^{a}}$ is a* nondominated solution* when there is no other solution $\vec{x^{b}}$ such that $\vec{F}(\vec{x^{a}})≺\vec{F}(\vec{x^{b}})$. Nondominated solution is also known as Pareto optimal solution.

**Definition 3**

The set of nondominated solutions of a MOO problem is known as* Pareto optimal set*, *P*.

**Definition 4**

The set of objective vectors with respect to *P* is known as the* Pareto front*, $PF=\{\vec{F}(\vec{x})\in R^{m}|\vec{x}\in P\}$. *PF* for a two-objective problem is illustrated as the black circles in Figure 1.

The motivation of MOO is to find as many nondominated solutions as possible according to the objective functions and constraints. However, it is possible to have different solutions which map to the same fitness value in objective space. Therefore, it will be more challenging to find more nondominated solutions.

## 3. Particle Swarm Optimisation

### 3.1. Original Particle Swarm Optimisation Algorithm

Particle swarm optimisation (PSO) is a population-based stochastic optimisation algorithm introduced by Kennedy and Eberhart [17]. This algorithm finds an optimal solution using a method inspired by the social behaviour of birds flocking and fish schooling. In the PSO algorithm, an individual is known as a particle, and it holds the possible solution to the optimisation problem, given its position. A particle explores the search space, looking for a better solution with respect to the objective functions defined by the optimisation problem. The search process requires the particle to compare its current position with the best positions that it and the whole swarm have found, so that all particles collaborate with each other.

The PSO algorithm is shown in Algorithm 1. Consider a minimisation problem in which a swarm of *I* particles are flying around in an *N*-dimensional search space, each with a position *p*_*n*_^*i*^  (*i* = 1,2,…, *I*; *n* = 1,2,…, *N*) representing the possible solution. At initialization stage, all particles are randomly positioned in the search space with random velocity, *v*_*n*_^*i*^(*t*). Subsequently, the objective fitness $\vec{F^{i}}(t)$ of each particle is evaluated based on the objective function for *p* ^*i*^(*t*). After that, the particle's best position, *p*Best^*i*^(*t*), is set to its initial position. Additionally, the swarm's best position, *g*Best(*t*), is the best *p*Best^*i*^(*t*) among all particles, as in (2), where *S* is the swarm of particles

(2) $$\begin{array}{c} g\text{Best}=\left\{p\text{Bes}{\text{t}}^{i}\in S\;|\;f\left(p\text{Bes}{\text{t}}^{i}\right)=\min f\left(\forall p\text{Bes}{\text{t}}^{i}\in S\right)\right\}. \end{array}$$

In the search process, the algorithm will iterate until the maximum number of iterations is reached. Within an iteration, the velocity and position of each particle are updated using (3) and (4), respectively,

(3) vni(t+1)=χ[ωvni(t)+c1r1(pBestni−pni(t))+c2r2(gBestn−pni(t))],

(4) $$\begin{array}{c} p_{n}^{i}\left(t+1\right)=p_{n}^{i}\left(t\right)+v_{n}^{i}\left(t+1\right), \end{array}$$

where *χ* is the constriction factor and *ω* is the inertia weight. The *r*_1_ and *r*_2_ are both random numbers ranging from zero to one. The *c*_1_ and *c*_2_ are the cognitive and social constants, respectively, which control the attraction of the *p*Best^*i*^(*t*) and *g*Best(*t*). Then, the $\vec{F^{i}}(t)$ for each particle is evaluated again. After updating the fitness, the new position of particle *i* is compared with *p*Best^*i*^(*t*), and the more optimal of the two is saved as *p*Best^*i*^(*t*). Next, the *g*Best(*t*) is updated as well with the best among all *p*Best^*i*^(*t*), as in (2). When the search process ended, the *g*Best(*t*) will then represent the best solution found for the problem by this algorithm.

### 3.2. Vector Evaluated Particle Swarm Optimisation Algorithm

The VEPSO algorithm, introduced by Parsopóulos and Vrahatis [1], uses the multiswarms concept from the VEGA algorithm [9]. Each swarm optimises one objective function using the *g*Best(*t*) from another swarm. In the VEPSO algorithm, the *p*Best^*i*^(*t*) which has the best fitness with respect to the *m*th objective is the *g*Best(*t*) for the *m*th swarm, as in (5)

(5) gBestm={pBesti∈Sm ∣ fm(pBesti)=min⁡fm(∀pBesti∈Sm)}.

**Figure 2:**
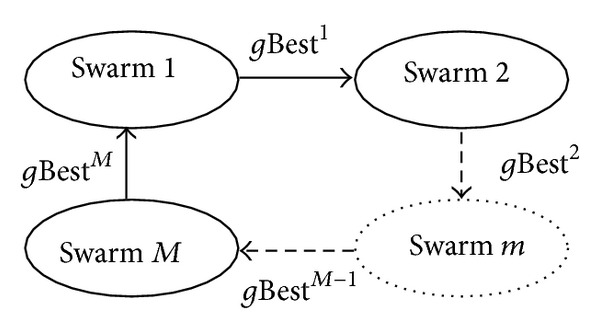
The best position found by the swarms, shared between all swarms.

The flow of the VEPSO algorithm is given as in Algorithm 2. For problem with *M* objective functions, VEPSO algorithm is similar to that of the PSO but some processes are repeated for all *M*-swarm and nondominated solutions are recorded in an archive. However, the velocity update is reformulated and it is given in (6). Note that the particles in the *m*th swarm will fly using *g*Best^*k*^(*t*) where *k* is defined by (7). The sharing of *g*Best(*t*) between swarms is illustrated in Figure 2:

(6) vnmi(t+1)=χ[ωvnmi(t)+c1r1(pBestnmi−pnmi(t))+c2r2(gBestnk−pnmi(t))]

(7) $$\begin{array}{c} k=\{\begin{array}{cc} M, & m=1\\ m-1, & \text{otherwise}. \end{array} \end{array}$$

The nondominated solutions are recorded in an archive after the objective functions are evaluated. In the recording process, the fitness $\vec{F^{i}}(t)$ of each particle is compared to all others, before it is compared to the nondominated solutions in the archive, using the* Pareto optimality* criterion, so that the archive only contains nondominated solutions. At the end of the computation, all nondominated solutions are the possible solutions to the problem.

### 3.3. The Improved VEPSO Algorithm by Incorporating Nondominated Solutions

**Figure 3:**
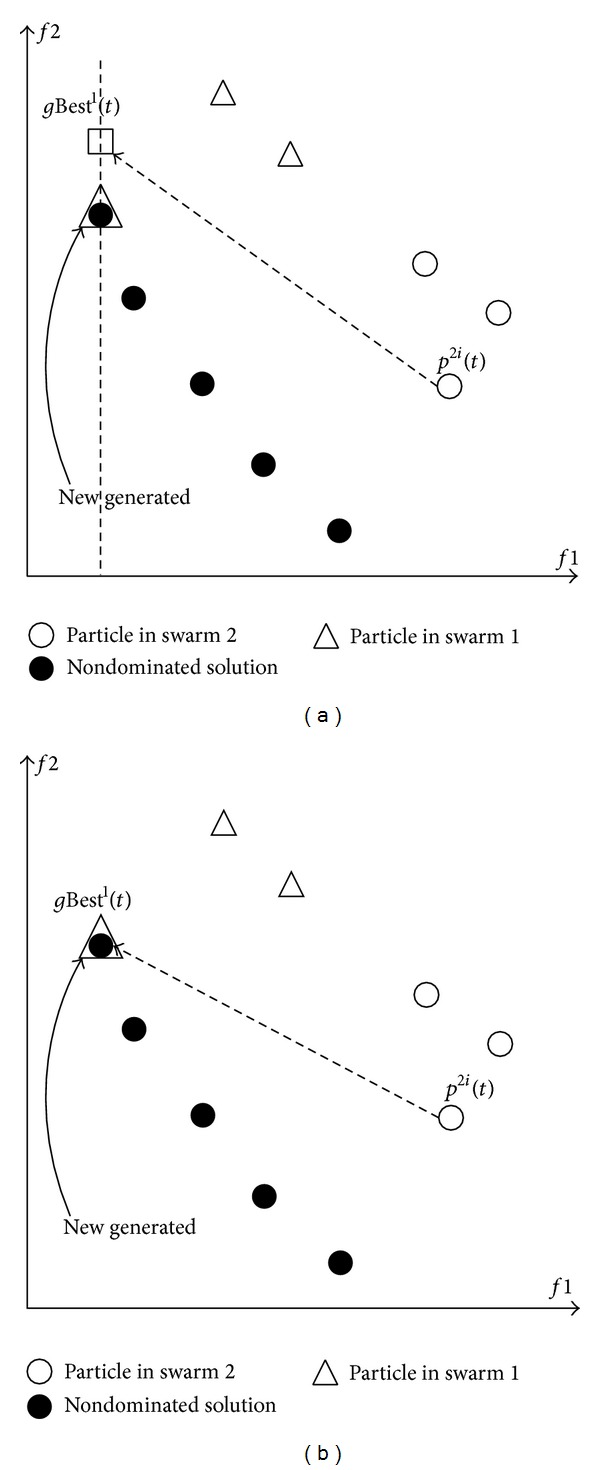
(a) Particles guided by the best solution from the other swarm (b) Particles guided by a nondominated solution with respect to another swarm.

In the search process of conventional VEPSO, as in Figure 3(a), particles from a swarm are optimised using the *g*Best^*m*^(*t*) from another swarm that has the best fitness at the objective function optimised by the other swarm. However, based on the velocity update of conventional VEPSO in (5), the *g*Best^*m*^(*t*) is not updated unless there is a *p*Best^*mi*^(*t*) that has better fitness than that at the *m*-objective. Consequently, in a two-objective MOO problem, the *g*Best^1^(*t*) of the first swarm is not updated when particle in the first swarm has found a solution with equal fitness at the first objective and better fitness at the second objective. Thus, particles from the second swarm will be guided toward the *g*Best^1^(*t*).

Due to this limitation, Lim et al. [10] have introduced an improved VEPSO algorithm by incorporating nondominated solutions (VEPSOnds). In VEPSOnds, as specified by (8), the *g*Best^*m*^(*t*) is still the solution with best fitness at *m*-objective function but is selected from the set of nondominated solutions and not from all *p*Best^*mi*^(*t*) of the *m*-swarm

(8) $$\begin{array}{c} g\text{Bes}{\text{t}}^{m}=\left\{X\in P\;|\;f_{m}\left(X\right)=\min f_{m}\left(\forall X\in P\right)\right\}, \end{array}$$

where *X* is a nondominated solution and *P* is the set of nondominated solutions in the archive.

This improvement is illustrated in Figure 3(b) where the *g*Best^*m*^(*t*) is always the best solution with respect to *m*-objective function because the other objective functions are considered as well. Hence, particles from the second swarm can converge faster towards the *g*Best^1^(*t*), which is a nondominated solution. As a result, better quality of Pareto front is obtained. From an algorithm perspective, the VEPSOnds is similar to the conventional VEPSO except that (5) in Algorithm 2 is replaced with (8).

### 3.4. The Improved VEPSO Using Multiple nondominated Leader

Based on the results of VEPSOnds [10], this algorithm suffers weak performance in obtaining solutions that has a weak diversity performance where the solution distributions along the Pareto front are not well distributed. Besides, in comparison to other state-of-the-art MOO algorithm, the VEPSOnds also has a problem in convergence where the obtained solution is far distant from the Pareto front. This weak performance could possibly be caused by the fact that particles in each swarm are guided by one *g*Best^*m*^(*t*) only so the obtained solutions do not well diverse to the other objective functions.

**Figure 4:**
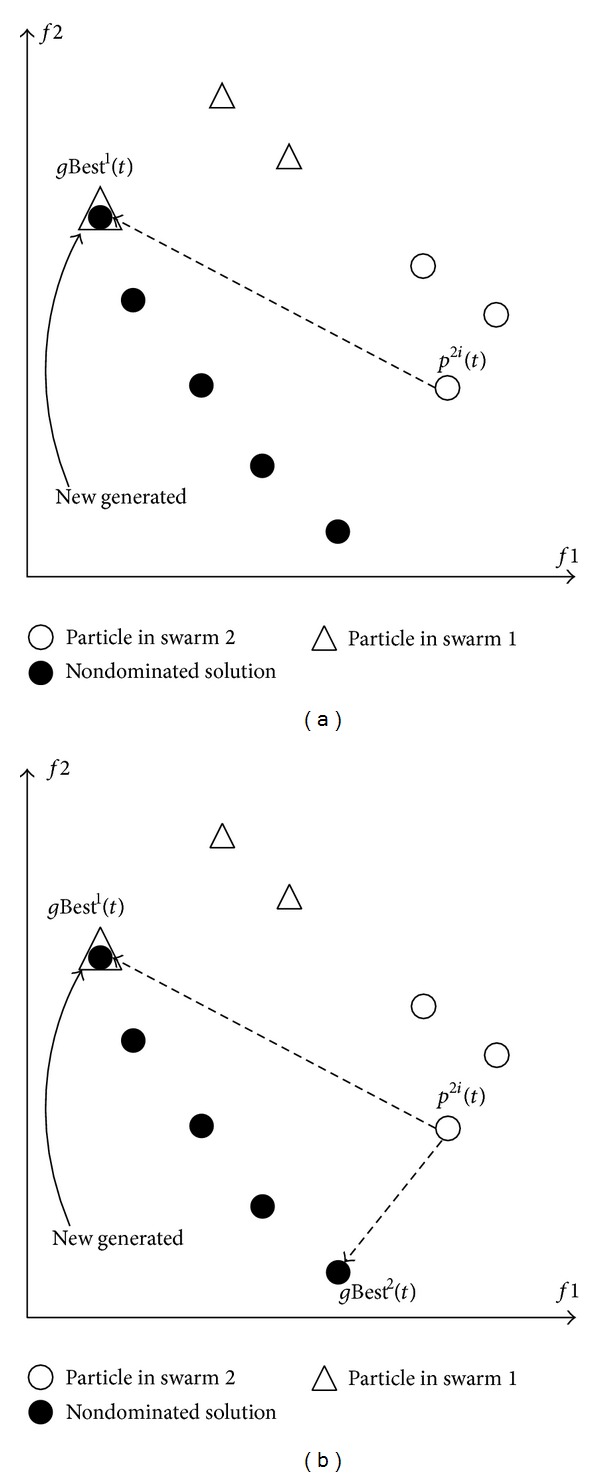
(a) A particle is guided based on *g*Best^1^(*t*). (b) A particle is guided based on *g*Best^1^(*t*) and *g*Best^2^(*t*).

Thus, the use of nondominated solutions to enhance the VEPSO algorithm can be further improved by the use of multileader concept in this work. According to (6), which is the velocity equation of the VEPSO, the particles of a swarm are guided by its *p*Best(*t*) and another swarm's *g*Best(*t*). For example, as shown in Figure 4(a), the particles from the second swarm optimise the second objective function using *g*Best^1^(*t*) only, which may not be the solution that has the best fitness with respect to the second objective function. Thus, this original mechanism of VEPSO may limit the convergence rate of the algorithm. Therefore, an improved VEPSO algorithm is proposed by including *g*Best^2^(*t*) as additional guidance to optimise both objective functions, as shown in Figure 4(b).

Hence, the general velocity equation of this improved VEPSO is formulated as in (9)

(9) vnmi(t+1)=χ[ωvnmi(t)+c1r1(pBestnmi−pnmi(t))+∑q=1Mc2qr2q(gBestnq−pnmi(t))],

where for each *q*,  *c*_2_^*q*^, and *r*_2_^*q*^ are independent constant and random values, respectively. In addition, from (9), as compared to the improved VEPSO at previous section, the particles will search toward the nondominated solutions which located at different end of the Pareto front. Therefore, the diversity performance of the algorithm is expected to be better as the search area is wider, rather than a single point.

Because the improved VEPSO algorithm uses multiple nondominated solutions as particle guides, or leaders, this algorithm is called VEPSO using multiple nondominated leaders (VEPSOml). Also, a polynomial mutation mechanism from NSGA-II [14] is used to modify particle positions at some probability. By mutating the position of some particles out of the locally optimal solution, this mechanism broadens the search for a globally optimal solution. In this study, the position of one out of every fifteen particles is mutated in the algorithm. Therefore, the complete VEPSO algorithm using multiple nondominated leaders is shown in Algorithm 3.

## 4. Experiment

### 4.1. Performance Measure

MOO algorithms face difficulty in converging to and distributing the nondominated solutions over the true Pareto front, *PF*_*t*_. Hence, the algorithm performance is measured by the quality of the obtained Pareto front, *PF*_*o*_. Several performance measures are used for comparison to highlight any improvement in the proposed algorithm.

The number of solutions (NS) measured will calculate the total number of nondominated solutions found by an algorithm. The best algorithm, by this measure, gives the most nondominated solutions. A more advanced measure uses the generalized distance (GD) [18], which is a popular measure of convergence [14]. This performance measure first evaluates the average distance between the true Pareto front and the one obtained by the algorithm. Equation (10) is used to compute the average distance, with a smaller value corresponding to a better performance. Then, the minimum distance of a nondominated solution from the true Pareto front is calculated using (11)

(10) GD=(∑q=1||PFo||dqM)1/M||PFo||

(11) dq=min⁡1≤g≤||PFt||∑m=1M(PFomq−PFtmg)2.

In addition, SP [14] is a commonly used measure of the diversity performance, or the distribution of nondominated solutions [14] is used. Equations (12), (13), and (14) evaluate the diversity performance, as measured by SP. The *d*_*f*_ and *d*_*l*_ are the Euclidean distances between the boundary solution and the nondominated solutions returned by the algorithm and the true Pareto front, respectively. The Euclidean distance between two solutions can be calculated using (13). Thus, SP actually measures the average distance of one solution and of the next solution to all nondominated solutions returned by the algorithm as well as two boundary solutions in the true Pareto front. Hence, it is desirable that the Pareto front returned by the algorithm produces a small SP:

(12) $$\begin{array}{c} \text{Spread}=\frac{d_{f}+d_{l}+\sum_{q=1}^{||PF_{o}||-1}\left|d_{q}-\bar{d}\right|}{d_{f}+d_{l}+\bar{d}\left(||PF_{o}||-1\right)}, \end{array}$$

(13) $$\begin{array}{c} d_{q}=\sqrt{{\left(P{F_{o}^{1}}_{q}-{PF_{o}^{1}}_{q+1}\right)}^{2}+{\left(P{F_{o}^{2}}_{q}-{PF_{o}^{2}}_{q+1}\right)}^{2}}, \end{array}$$

(14) $$\begin{array}{c} \bar{d}=\frac{\sum_{q=1}^{||PF_{o}||-1}d_{q}}{||PF_{o}||-1}. \end{array}$$

**Figure 5:**
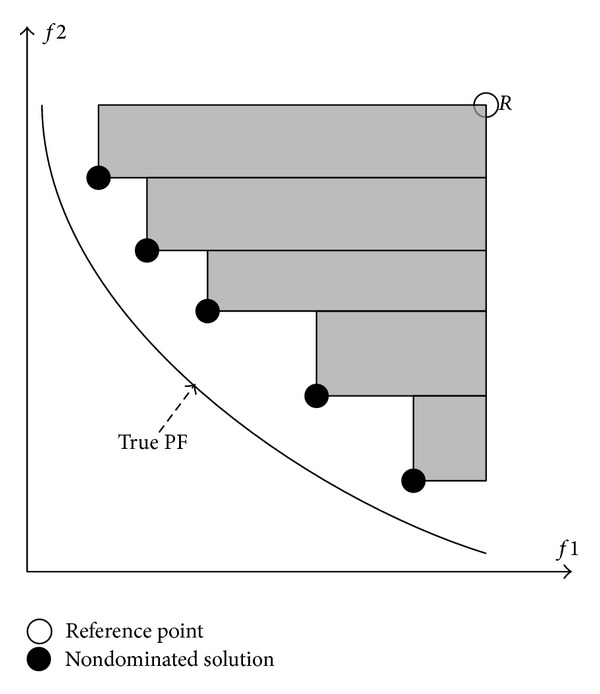
Hypervolume measure with area covered by nondominated solutions and reference point.

Additionally, the hypervolume (HV) [19] measures the area (in a two-objective problem) or the volume between a reference point, *R* and the Pareto front with respect to the nondominated solutions obtained by the algorithm, as illustrated in Figure 5. Thus, it is desirable that the Pareto front returned by the algorithm produces a large HV.

### 4.2. Test Problems

Because different features in MOO problems are responsible for decreasing the likelihood of obtaining Pareto front with good convergence and diversity, the standard test functions with well-defined true Pareto fronts are important for testing optimisation algorithms. Five test functions from Zitzler et al. [20] (ZDT) are used here for this reason. The ZDT test problems have two objectives and are formulated with one feature in each problem. ZDT5 is not used because it is binary coded, whereas this work focuses on real-value problems. During testing, the GD, SP, and HV measure require the true Pareto front for the ZDT test problems, the standard database generated by jMetal (http://jmetal.sourceforge.net/problems.html) is used for this purpose. Additionally, all test problems used here are set up as recommended by [20].

### 4.3. Evaluation of VEPSO Algorithms

The performance comparison between conventional VEPSO, VEPSOnds, and VEPSOml is conducted without the use of polynomial mutation as to clarify that the polynomial mutation is not the sole reason for any performance improvement. Thus, this experiment compares the conventional VEPSO and the VEPSOnds without mutation against two different variations of VEPSOml: VEPSOml1 is the VEPSOml without mutation and VEPSOml2 is the VEPSOml with mutation, respectively.

All improved VEPSO algorithms are compared to the conventional VEPSO algorithm. Hence, similar parameters are used for all experimented algorithms which are listed in Table 1. In addition, the archive size is set to 100 solutions and is controlled by removing the nondominated solutions with the smallest crowding distance [14]. Each test problem is simulated for 100 runs on each algorithm to obtain statistical results for a fair comparison because the convergence and diversity performance varies in each run.

Table 2 lists the performance of each algorithm on the ZDT1 test problem. In the NS measure, the number of nondominated solutions significantly increases in all improved algorithms. Under the GD measure, VEPSOnds performs approximately 10 times better than conventional VEPSO. However, under the same measure, VEPSOml1 shows a more dramatic improvement, performing approximately 100 times better than VEPSO, as the concept of multiple nondominated leaders shows its benefit in finding more accurate solutions. Additionally, when the polynomial mutation is included, as in VEPSOml2, the GD performance improved much better at about 600% as compared to the conventional VEPSO. Under the SP measure, VEPSOnds also gives a significant improvement in performance. Meanwhile, the VEPSOml1 and VEPSOml2 show significant improvement in diversity performance as compared to the VEPSOnds. This shows the significance of using more than one nondominated solution which diversify the search toward the nondominated solutions at different end. The above mentioned improvements are supported by the higher HV measures when compared to the conventional VEPSO, which indicates that they return better Pareto fronts.

**Figure 6:**
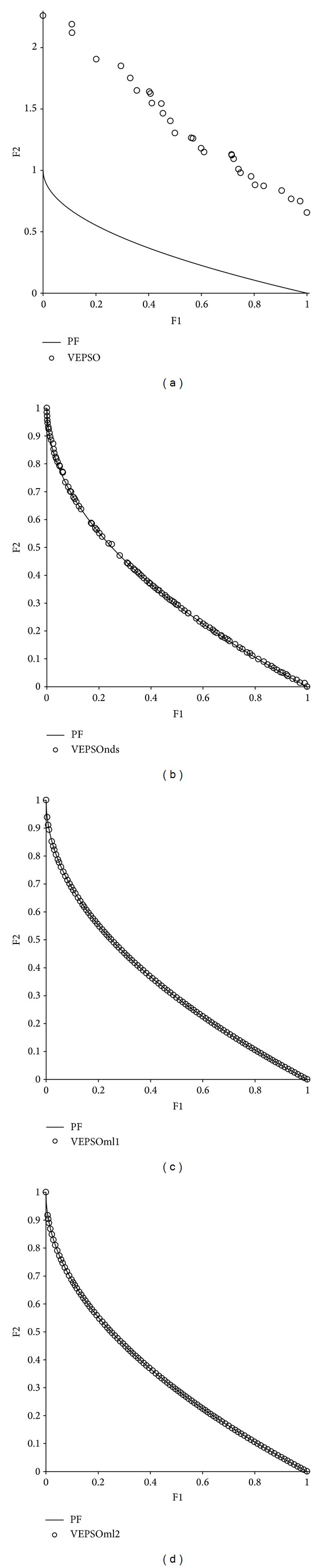
Plot of nondominated solutions returned by each algorithm for the ZDT1 test problem.

Figure 6 shows plots of the nondominated solutions with the best GD measure returned by each algorithm tested on ZDT1. From the first plot, it is clear that the nondominated solutions obtained by VEPSO are far away from the true Pareto front, which explains the poor performance of this algorithm for this test problem. In addition, the nondominated solutions are distributed unevenly, and so VEPSO has a larger SP value. Meanwhile for all the improved VEPSO algorithms, their nondominated solutions fall very close to the true Pareto front. However, VEPSOnds produces a distribution of nondominated solutions that contain empty spaces along the true Pareto front, which results in higher SP value as compared to the other improved VEPSO algorithms.

Table 3 lists the performance of the algorithms on the ZDT2 test problem. The average number of nondominated solutions found by VEPSOnds1 slightly improves over the number found by VEPSO, but VEPSOnds2, VEPSOml1, and VEPSOml2 greatly improve over VEPSO by this same measure. Similarly, by the GD measure, VEPSOnds1 shows a small improvement, whereas VEPSOnds2 and VEPSOml1 show a larger improvement over the performance of VEPSO. In the same measure, VEPSOml2 shows a more significant improvement over the VEPSO and all other improved algorithms. Additionally, by the SP measure, VEPSOnds1 shows negligible improvement, whereas VEPSOnds2 shows a significant improvement over the performance of VEPSO. Besides, with the use of multileader, VEPSOml shows much better diversity performance than both the VEPSOnds. Finally, by the HV measure, VEPSO was unable to produce any hypervolume because its nondominated solutions are worse than the reference point, *R*. On the other hand, all improved algorithms are able to create a hypervolume, especially the VEPSOml2 which produce the largest hypervolume.

**Figure 7:**
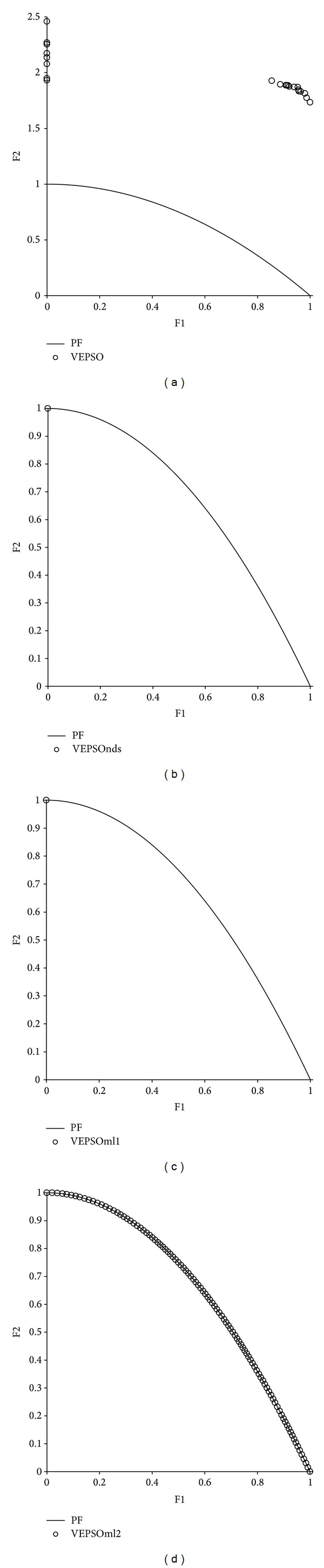
Plot of nondominated solutions returned by each algorithm for the ZDT2 test problem.

Figure 7 displays the nondominated solutions, plotted for each the best GD measure obtained for each algorithm using the ZDT2 test problem. The first plot shows that VEPSO returns nondominated solutions that are far from the true Pareto font and poorly distributed. Although VEPSOnds and VEPSOml1 return a low GD measure, the number of nondominated solutions is found to have low value, which is clearly displayed in the second and third plots, respectively, of Figure 6. In fact, there is only one nondominated solution found by both algorithms which falls exactly on the true Pareto front and yields a GD value of zero. On the other hand, the fourth plot of Figure 6 shows that VEPSOml2 returns the nondominated solutions that converge nicely and are well distributed over the true Pareto front. Besides, the nondominated solutions found by VEPSOml2 distributed evenly which yield a good SP value.

Table 4 lists the performance of the algorithms on the ZDT3 test problem. All improved VEPSO algorithms are able to find more nondominated solutions than the conventional VEPSO algorithm. In addition, the performances of the improved VEPSO algorithms, with respect to convergence, improve on conventional VEPSO, while VEPSOml2 shows the greater improvement. However, by the SP measure, the VEPSOnds algorithm performs worse than the conventional VEPSO algorithm. However, although the SP value of the conventional VEPSO algorithm is better, the superior convergence of the VEPSOnds algorithm maintains its performance advantage. In contrast, both improved VEPSO algorithm using multiple nondominated leaders show better SP measure than the conventional VEPSO, which strengthen the hypothesis that using multiple nondominated leaders will improve diversity performance. In addition, the HV value of the conventional VEPSO algorithm is smaller than of all improved algorithms which suggest that the improved algorithms have better performance.

**Figure 8:**
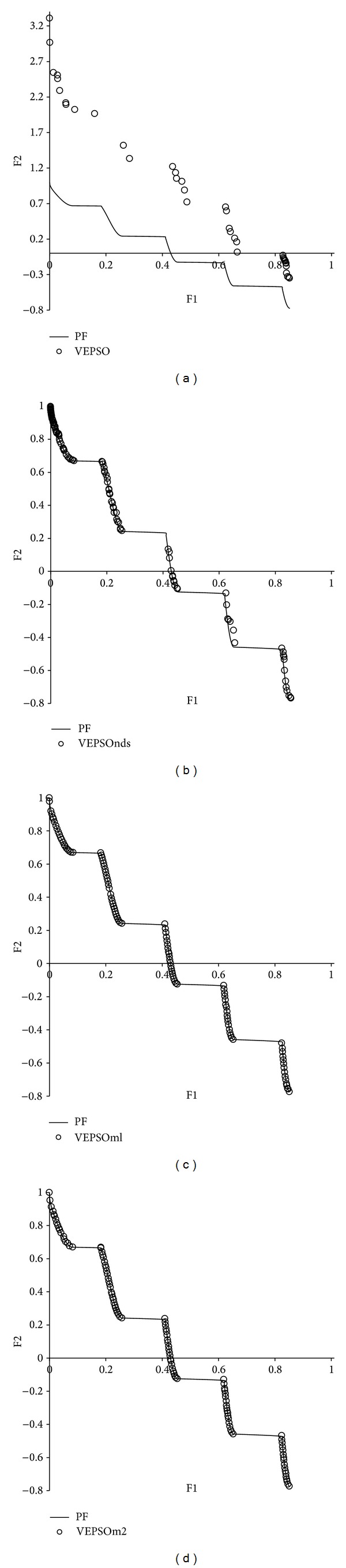
Plot of nondominated solutions returned by each algorithm for the ZDT3 test problem.

Figure 8 displays the nondominated solutions, plotted for the best GD measure obtained for each algorithm using the ZDT3 test problem. The nondominated solutions returned by the conventional VEPSO algorithm were distributed equally but not well converged with respect to the true Pareto front. On the other hand, the nondominated solutions from all improved VEPSO algorithms are well converged with respect to the true Pareto front. However, the nondominated solutions returned by the VEPSOnds algorithm are denser at the upper left of the Pareto front, which causes the increase in its SP value. In contrast, the nondominated solutions obtained by both VEPSOml algorithms are equally distributed over the Pareto front and yield better SP value.

Table 5 lists the performance of the algorithms on the ZDT4 test problem. The average number of nondominated solutions obtained by VEPSO is relatively low, while all improved VEPSO algorithms found most of the solutions. In this test, the conventional VEPSO algorithm produced a very large GD value due to the multimodality feature in the test problem, and so the improved VEPSO algorithms clearly performed better in this respect. However, the diversity performance of nondominated solutions returned by conventional VEPSO is small compared to the VEPSOnds algorithm. Once again, the use of multiple nondominated leaders in VEPSO algorithms could diversify the search and result in better diversity performance. Additionally, all algorithms produce a hypervolume from the reference point, and all improved algorithms return larger HV values than the conventional algorithm.

**Figure 9:**
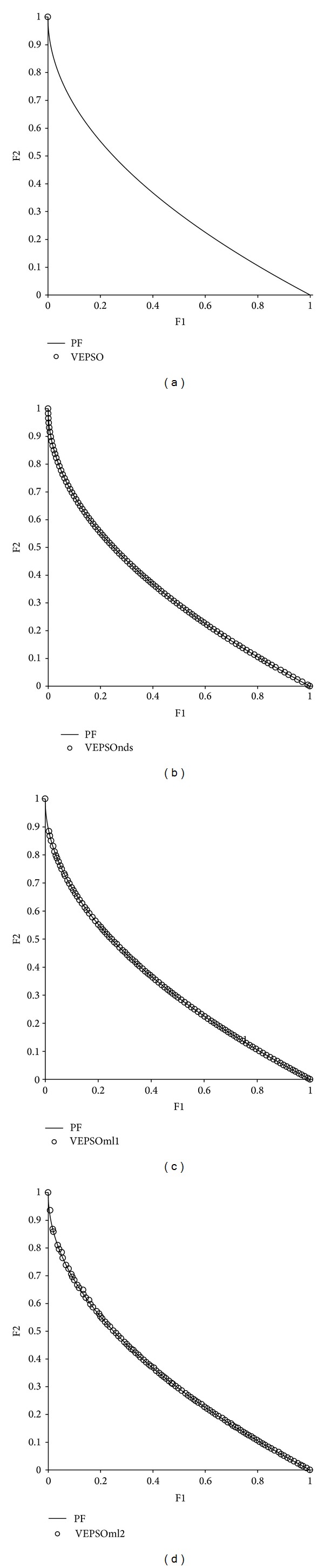
Plot of nondominated solutions returned by each algorithm for the ZDT4 test problem.

Figure 9 displays the nondominated solutions, plotted for the best GD measure obtained for each algorithm using the ZDT4 test problem. The first plot shows that VEPSO converges to the Pareto front but only manages to obtain a single nondominated solution. The VEPSOnds algorithm not only converges to the Pareto front but also returns a diverse set of nondominated solutions. On the other hands, both VEPSOml also returned the nondominated solutions with good convergence but they are not well distributed as compared to the VEPSOnds, in this case. Thus, the VEPSOnds shows better HV value as compared to the VEPSOml.

Table 6 lists the performance of the algorithms on the ZDT6 test problem. All algorithms find a similar number of nondominated solutions. In the GD measure, all algorithms are capable of returning the nondominated solutions that converge well to the Pareto front. On the other hand, both VEPSOml1 and VEPSOml2 algorithms outperform the conventional VEPSO and VEPSOnds algorithm in the GD measure. In addition, the SP and HV values for each algorithm are similar. However, the VEPSOml2 algorithm shows superiority in getting the minimum SP value and average HV value.

**Figure 10:**
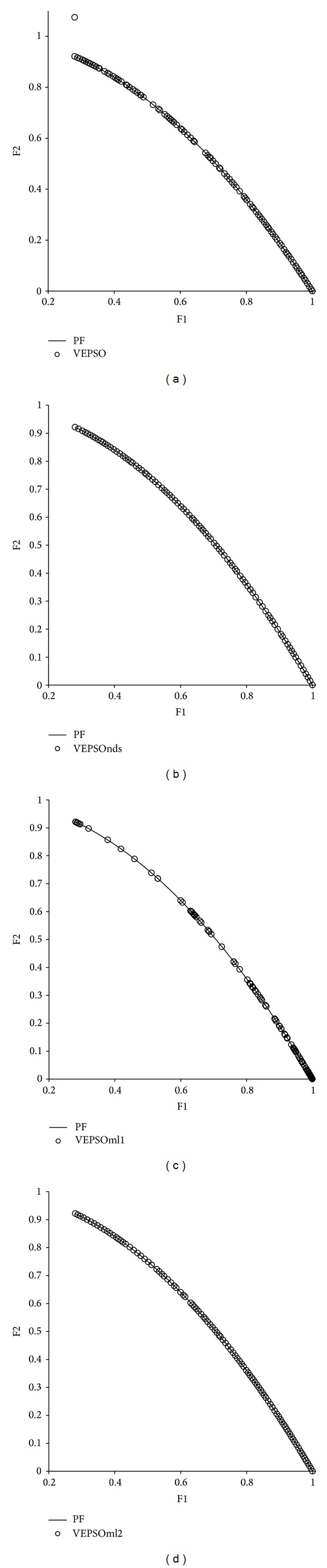
Plot of nondominated solutions returned by each algorithm for the ZDT6 test problem.

As can be predicted from the similar quantitative performance of the algorithms on ZDT6, the plot of nondominated solutions returned by each algorithm is very similar, especially in convergence performance, as shown in Figure 10. The plots do show that VEPSO has slightly less diversity compared to VEPSOnds and VEPSOml2 because of some small gaps in coverage along the middle of the Pareto front. On the other hand, the VEPSOml1 shows weak distribution of nondominated solutions over the Pareto front. In contrast, the nondominated solutions found by VEPSOnds and VEPSOml2 completely cover the true Pareto front and are spaced out equally.

As seen from the results of all the test problems, the VEPSO algorithms using multiple nondominated leaders shows more improvement in terms of convergence and diversity of the nondominated solutions found than the VEPSOnds. The additional leader, specifically the nondominated solution with respect to the objective function optimised by a swarm, not only guides the particles to optimise the objective function with respect to the swarm. It also increased the search area because all leaders used to guide the particles are located at the different end of the Pareto front.

### 4.4. Analysis of the Number of Particles

**Figure 11:**
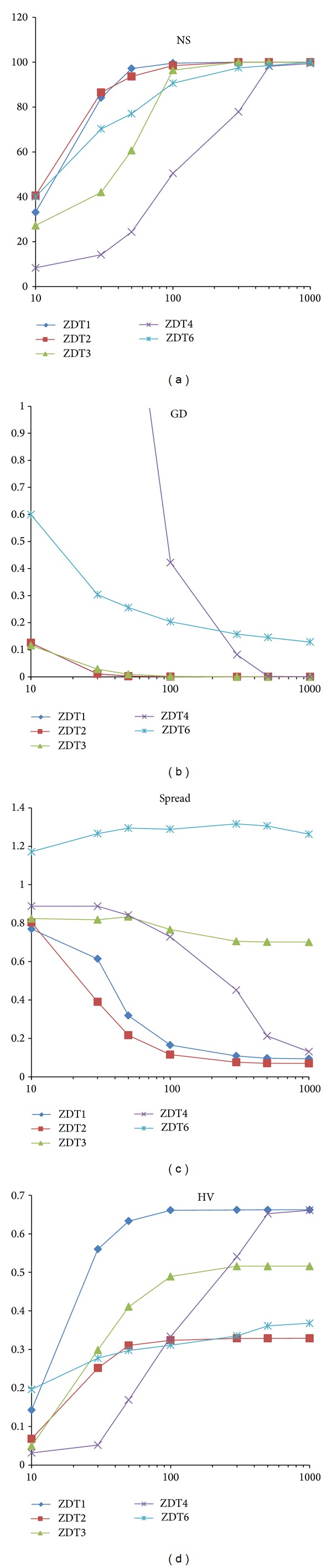
Plots of the performance measures versus numbers of particles. (a) Number of solutions. (b) Generational distance. (c) Spread. (d) Hypervolume.

This experiment analysed the performance of the VEPSOml2 algorithm with various numbers of particles. Similar parameters from the previous experiment were used except for the total number of particles as it is equally divided into two swarms; the total number of particles was varied to be 10, 30, 50, 100, 300, 500, and 1000.  Figure 11 shows plots of the performance measures for each benchmark problem against the total number of particles.

The VEPSOml2 algorithm performance improved as the number of particles increased. The performance of the VEPSOml2 algorithm was sufficient when there were 100 particles computed for 250 iterations, which corresponds to 25000 function evaluations. However, the performance of the VEPSOml2 algorithm exhibited better results when the total number of particles was increased. Unfortunately, when the number of particles is increased, the algorithm requires more computational effort to solve the problem.

### 4.5. Analysis of the Number of Iterations

**Figure 12:**
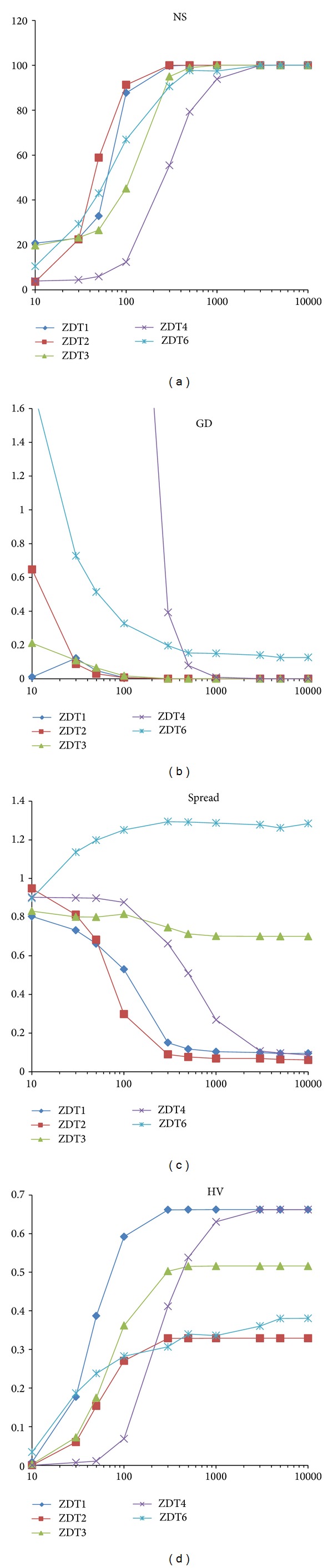
Plots of the performance metrics for various numbers of iterations. (a) Number of solution. (b) Generational distance. (c) Spread. (d) Hypervolume.

This experiment investigated the performance of VEPSOml2 for various numbers of iterations. The number of iterations was fixed to be 10, 30, 50, 100, 300, 500, 1000, 3000, 5000, and 10 000. Meanwhile, the other parameters were kept the same as in the previous experiment except that the number of particles, which were divided equally among swarms, was fixed to 100 divided equally between all swarms.  Figure 12 plots the performance measures for each benchmark problem against the number of iterations.

As expected, the performance of VEPSOml2 is improved when the number of iterations was increased. When 100 particles were used, the VEPSOml2 algorithm started to yield acceptable results when there were 500 iterations, which is equivalent to 50000 function evaluations. However, if computational cost is not critical, the VEPSOml2 algorithm could use 3000 iterations because the performance saturated after this value.

### 4.6. Benchmarking with the State-of-the-Art Multiobjective Optimisation Algorithms

For benchmarking, the VEPSOml2 algorithm was compared to four other state-of-the-art MOO algorithms: nondominated sorting genetic algorithm-II (NSGA-II) [14], strength Pareto evolutionary algorithm 2 (SPEA2) [21], archive-based hybrid scatter search (AbYSS) [22], and the speed-constrained multiobjective PSO (SMPSO) algorithm [23]. All algorithms only computed 25000 function evaluations, and the archive size was set to 100 for fair comparison. The population size for NSGA-II was set to 100 for optimisation. The Simulated Binary Crossover (SBX) operator was used with crossover probability  *p*_*c*_ = 0.9. The polynomial mutation [24] operator was also used with mutation probability *p*_*m*_ = 1/*N*. Meanwhile, the distribution indices for both operators were set to *μ*_*n*_ = *μ*_*m*_ = 20. The parameters in SPEA2 were set the same as in NSGA-II. The population size for AbYSS was set to 20 and the pairwise combination parameters *RefSet*_1_ and *RefSet*_2_ were both set to 10. In addition, the polynomial mutation parameters in AbYSS were also set similarly as in NSGA-II and SPEA2. Finally, SMPSO was set to have a population size of 100 particles and a total number of iterations of 250. Moreover, the *r*_1_ = *r*_2_ = random[0.1,0.5], and the terms *c*_1_ = *c*_2_ = random[1.5,2.0]. The polynomial mutation [25] operator was also used in SMPSO with *p*_*m*_ = 1/*N* and *μ*_*m*_ = 20.

The performance measures for the ZDT1 problem for all algorithms are listed in Table 7. The average number of solutions obtained by the VEPSOml2 was very similar to the other algorithms. Although VEPSOml2 algorithm had a GD measure approximately twice as large as those of the other algorithms, its minimum GD was still the smallest among them. However, the SP was, on average, better than NSGA-II. Interestingly, the HV measure of VEPSOml2 was as good as those of the other algorithms.

Table 8 presents the performance measure of the algorithms for the ZDT2 problem. The VEPSOml2 was sufficiently competitive at obtaining a reasonable number of solutions. In the GD measure, on average, the VEPSOml2 algorithm was as good as the other algorithms, but SMPSO had greater performance. Surprisingly, the VEPSOml2 algorithm was able to obtain a better minimum GD measure than the SMPSO algorithm. Additionally, the SP measure of the VEPSOml2 algorithm was better than those of the other algorithms except SMPSO. All algorithms had similar HV values, but VEPSOml2 yielded the best HV performance.

The performance measures for the ZDT3 problem for all algorithms are listed in Table 9. Both SMPSO and VEPSOml2 were unable to obtain the maximum number of solutions consistently for all 100 runs but still yielded solutions within a reasonable range. Noticeably, the average GD measure for VEPSOml2 was the largest among all algorithms. However, the diversity for VEPSOml2 was similar to that of the others. Moreover, although the HV value of VEPSOml2 was the smallest, it still yielded a very large HV.

Table 10 presents the performance measures for the algorithms for the ZDT4 problem. VEPSOml2 faced great challenges from the multiple local optima featured in this problem, where it cause the algorithm to obtain a very small number of solutions. Additionally, the convergence and diversity of VEPSOml2 were bad, as indicated by the very large GD and SP values. As expected, the HV performance was also very poor because the multiple local optima feature is one of the natural weaknesses of PSO-based algorithms [26, 27].

Finally, the performance measures for the ZDT6 problem for all algorithms are listed in Table 11. VEPSOml2 algorithm was inconsistent in obtaining the maximum number of solutions. Moreover, the convergence and diversity measures for VEPSOml2 were significantly larger than those for the other algorithms. However, the VEPSOml2 algorithm was able to obtain the minimum GD value. Additionally, the HV performance for VEPSOml2 was relatively weak, on average, but its maximum HV value was the largest of all the algorithms.

An overall performance comparison for state-of-the-art algorithms against VEPSOml2 was investigated in this experiment. In some cases, the VEPSOml2 algorithm yielded better results than some of the other algorithms.

## 5. Conclusions

Most PSO-based MOO algorithms, including conventional VEPSO and VEPSOnds, only use one solution as the particle guide. Thus VEPSOml is proposed in this study where the particles are guided by multiple nondominated solutions while retaining the unique information shared between swarms that are inherent in conventional VEPSO.

Five ZDT test problems were used to investigate the performance of the improved VEPSO algorithm based on the measures of the number of nondominated solutions found, the* generational distance*, the* spread,* and the* hypervolume*. The proposed VEPSOml algorithm obtained a higher-quality Pareto front as compared to conventional VEPSO and VEPSOnds. The VEPSOml2 algorithm that included polynomial mutation has exhibited further improvement for most of the performance measures.

Using more nondominated solutions as particle guides yielded faster convergence performance improvements, especially for the ZDT1, ZDT2, and ZDT3 test problems. The use of more than one leader reduced the risk of trapping at local Pareto front. In future, the success of using two leaders motivates the investigation of the use of more than two leaders during the optimisation process.

## Figures and Tables

**Algorithm 1:**
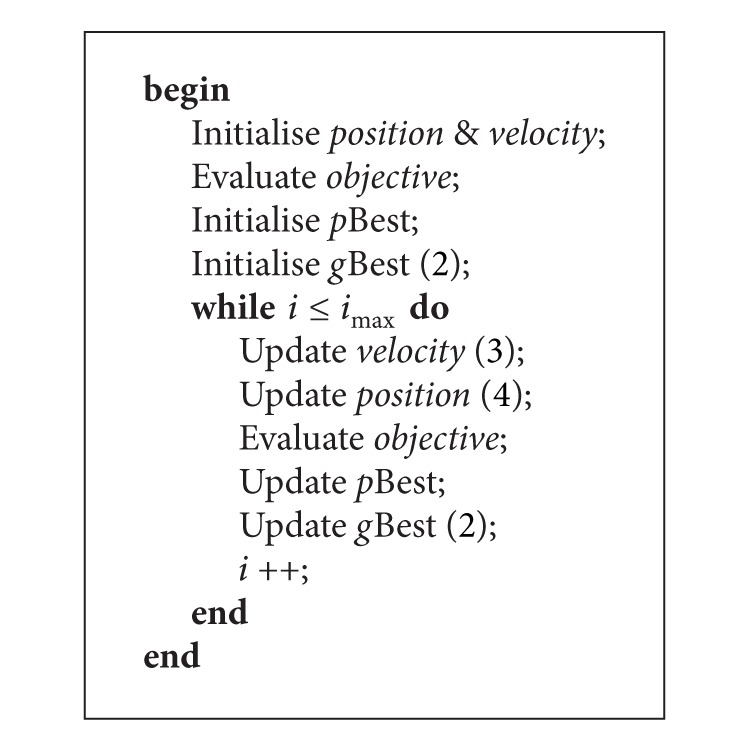
The PSO algorithm.

**Algorithm 2:**
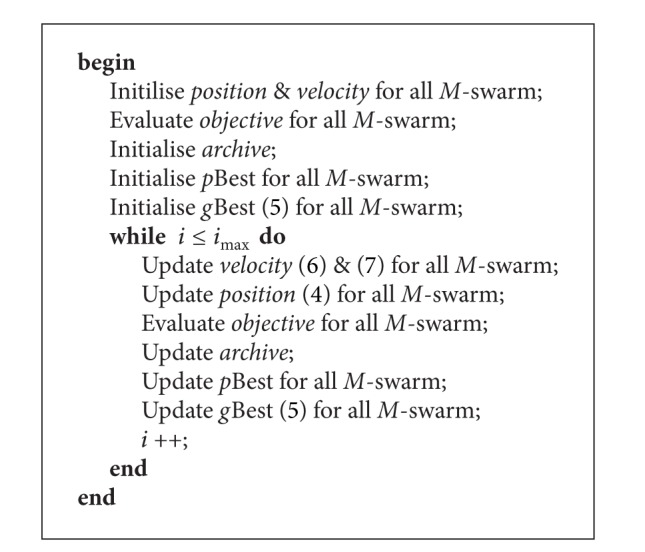
The VEPSO algorithm.

**Algorithm 3:**
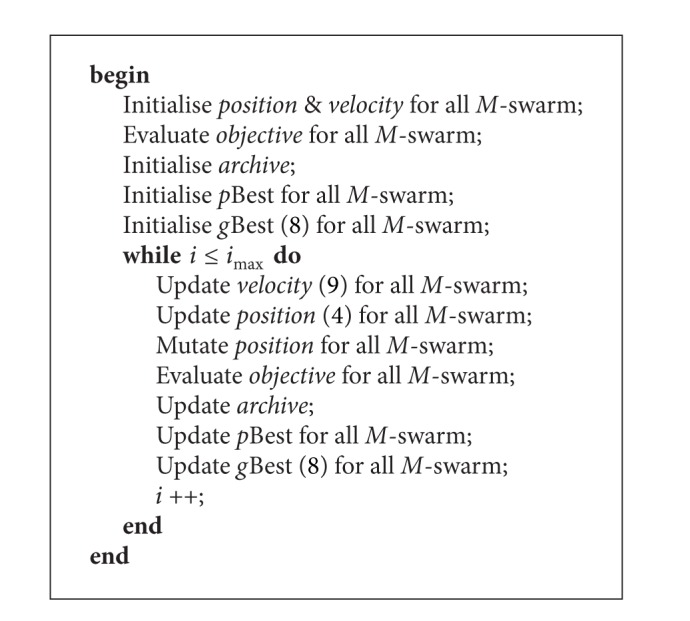
The VEPSO algorithm using multinondominated leaders.

**Table 1:** Algorithm parameters.

Parameter	Value
Function evaluation	25000 (based on paper [14])
(i) Number of swarm	2
(ii) Particle for each swarm	50
(iii) Iterations for each run	250

*c*_1_ and *c*_2_	Random [1.5, 2.5]

*ω*	Linearly degrade from 1.0 to 0.4

**Table 2:** Algorithm performance tested on ZDT1 problem.

Measure		VEPSO	VEPSOnds	VEPSOml1	VEPSOml2
NS	Ave.	30.220000	100.000000	99.490000	98.820000
	SD	5.697031	0.000000	3.942760	6.979595
	Min.	16.000000	100.000000	63.000000	47.000000
	Max.	44.000000	100.000000	100.000000	100.000000

GD	Ave.	0.295865	0.022637	0.002730	0.000497
	SD	0.051645	0.014201	0.006219	0.002213
	Min.	0.139491	0.000283	0.000045	0.000047
	Max.	0.432478	0.073477	0.031891	0.015598

SP	Ave.	0.834481	0.729350	0.212479	0.182157
	SD	0.039111	0.160298	0.149696	0.113453
	Min.	0.705367	0.322322	0.106082	0.109998
	Max.	0.917087	1.219625	0.738619	0.779572

HV	Ave.	0.001886	0.428153	0.628841	0.657830
	SD	0.010058	0.113432	0.078273	0.023359
	Min.	—	0.185313	0.283932	0.456556
	Max.	0.087426	0.659603	0.662065	0.662022

**Table 3:** Algorithm performance tested on ZDT2 problem.

Measure		VEPSO	VEPSOnds	VEPSOml1	VEPSOml2
NS	Ave.	8.070000	38.120000	91.090000	99.620000
	SD	6.356822	25.747131	28.474726	3.800000
	Min.	1.000000	1.000000	1.000000	62.000000
	Max.	24.000000	100.000000	100.000000	100.000000

GD	Ave.	0.766956	0.039653	0.005109	0.000152
	SD	0.324444	0.063791	0.010867	0.001009
	Min.	0.240509	0.000000	0.000000	0.000043
	Max.	1.679803	0.310345	0.028380	0.010144

SP	Ave.	0.944524	0.947356	0.267797	0.098572
	SD	0.065266	0.111963	0.315008	0.065826
	Min.	0.797757	0.695715	0.059578	0.064648
	Max.	1.080351	1.278655	1.000004	0.721104

HV	Ave.	—	0.137784	0.250495	0.328291
	SD	—	0.117596	0.127625	0.004182
	Min.	—	—	0.000000	0.286901
	Max.	—	0.311075	0.328807	0.328816

**Table 4:** Algorithm performance tested on ZDT3 problem.

Measure		VEPSO	VEPSOnds	VEPSOml1	VEPSOml2
NS	Ave.	35.150000	99.600000	95.710000	96.500000
	SD	6.853997	3.405284	11.528003	11.372037
	Min.	21.000000	66.000000	46.000000	49.000000
	Max.	53.000000	100.000000	100.000000	100.000000

GD	Ave.	0.173060	0.009607	0.002586	0.001456
	SD	0.031253	0.008293	0.003904	0.002533
	Min.	0.079595	0.000433	0.000153	0.000159
	Max.	0.276801	0.039481	0.017547	0.007328

SP	Ave.	0.871146	1.109448	0.761061	0.752151
	SD	0.043319	0.086041	0.056129	0.050459
	Min.	0.701884	0.902861	0.701924	0.703181
	Max.	1.001428	1.322024	0.934796	0.981492

HV	Ave.	0.004722	0.373133	0.476679	0.493073
	SD	0.021699	0.083015	0.060626	0.045211
	Min.	—	0.112859	0.289513	0.391275
	Max.	0.167359	0.506222	0.515919	0.515941

**Table 5:** Algorithm performance tested on ZDT4 problem.

Measure		VEPSO	VEPSOnds	VEPSOml1	VEPSOml2
NS	Ave.	6.610000	95.250000	82.730000	51.470000
	SD	3.920665	16.518967	30.304800	35.623864
	Min.	1.000000	15.000000	6.000000	4.000000
	Max.	21.000000	100.000000	100.000000	100.000000

GD	Ave.	5.062543	0.383646	0.231380	0.449095
	SD	3.167428	0.478535	0.841726	1.060986
	Min.	0.000000	0.000155	0.000062	0.000146
	Max.	13.350278	2.049212	7.013747	6.835452

SP	Ave.	0.858655	1.035510	0.572461	0.735715
	SD	0.147255	0.347336	0.286004	0.201246
	Min.	0.483073	0.077112	0.135264	0.269484
	Max.	1.236461	1.419225	1.139773	1.088971

HV	Ave.	0.228824	0.399914	0.357553	0.307568
	SD	0.188151	0.159971	0.281263	0.272435
	Min.	—	—	—	—
	Max.	0.573978	0.661941	0.661917	0.660309

**Table 6:** Algorithm performance tested on ZDT6 problem.

Measure		VEPSO	VEPSOnds	VEPSOml1	VEPSOml2
NS	Ave.	76.590000	78.040000	86.920000	88.590000
	SD	32.884891	26.684055	23.586368	23.600674
	Min.	11.000000	22.000000	25.000000	16.000000
	Max.	100.000000	100.000000	100.000000	100.000000

GD	Ave.	0.338537	0.260666	0.217503	0.217929
	SD	0.370336	0.158592	0.214344	0.263966
	Min.	0.001746	0.044137	0.031135	0.000482
	Max.	1.552521	0.709692	1.184075	1.316312

SP	Ave.	1.201796	1.276529	1.301493	1.273612
	SD	0.146782	0.083293	0.085611	0.186562
	Min.	0.492064	0.987981	0.931549	0.082405
	Max.	1.435395	1.437289	1.430321	1.439400

HV	Ave.	0.304584	0.303381	0.303676	0.315964
	SD	0.134813	0.102216	0.123985	0.121842
	Min.	0.000000	0.038143	0.000779	0.000001
	Max.	0.400964	0.400780	0.401403	0.401483

**Table 7:** Performance comparison based on ZDT1 test problem.

Measure		AbYSS	NSGA-II	SPEA2	SMPSO	VEPSOml2
NS	Ave.	100.000000	100.000000	100.000000	100.000000	98.820000
	SD	0.000000	0.000000	0.000000	0.000000	6.979595
	Min.	100.000000	100.000000	100.000000	100.000000	47.000000
	Max.	100.000000	100.000000	100.000000	100.000000	100.000000

GD	Ave.	0.000185	0.000223	0.000220	0.000117	0.000497
	SD	0.000035	0.000038	0.000028	0.000031	0.002213
	Min.	0.000125	0.000146	0.000154	0.000053	0.000047
	Max.	0.000343	0.000374	0.000400	0.000172	0.015598

SP	Ave.	0.105387	0.379129	0.148572	0.076608	0.182157
	SD	0.012509	0.028973	0.012461	0.009200	0.113453
	Min.	0.080690	0.282485	0.116765	0.056009	0.109998
	Max.	0.136747	0.441002	0.174986	0.099653	0.779572

HV	Ave.	0.661366	0.659333	0.659999	0.661801	0.657830
	SD	0.000269	0.000301	0.000301	0.000100	0.023359
	Min.	0.660267	0.658486	0.659347	0.661372	0.456556
	Max.	0.661724	0.659909	0.660629	0.661991	0.662022

**Table 8:** Performance comparison based on ZDT2 test problem.

Measure		AbYSS	NSGA-II	SPEA2	SMPSO	VEPSOml2
NS	Ave.	100.000000	100.000000	100.000000	100.000000	99.620000
	SD	0.000000	0.000000	0.000000	0.000000	3.800000
	Min.	100.000000	100.000000	100.000000	100.000000	62.000000
	Max.	100.000000	100.000000	100.000000	100.000000	100.000000

GD	Ave.	0.000131	0.000176	0.000182	0.000051	0.000152
	SD	0.000067	0.000066	0.000039	0.000003	0.000152
	Min.	0.000056	0.000093	0.000090	0.000044	0.000043
	Max.	0.000433	0.000707	0.000304	0.000060	0.010144

SP	Ave.	0.130425	0.378029	0.158187	0.071698	0.098572
	SD	0.090712	0.028949	0.027529	0.013981	0.065826
	Min.	0.080831	0.311225	0.118114	0.035786	0.064648
	Max.	0.833933	0.430516	0.365650	0.106749	0.721104

HV	Ave.	0.325483	0.326117	0.326252	0.328576	0.328291
	SD	0.023209	0.000297	0.000908	0.000077	0.004182
	Min.	0.096409	0.325278	0.318785	0.328349	0.286901
	Max.	0.328505	0.326696	0.327559	0.328736	0.328816

**Table 9:** Performance comparison based on ZDT3 test problem.

Measure		AbYSS	NSGA-II	SPEA2	SMPSO	VEPSOml2
NS	Ave.	100.000000	100.000000	100.000000	99.900000	96.500000
	SD	0.000000	0.000000	0.000000	0.904534	11.372037
	Min.	100.000000	100.000000	100.000000	91.000000	49.000000
	Max.	100.000000	100.000000	100.000000	100.00000	100.000000

GD	Ave.	0.000193	0.000211	0.000230	0.000203	0.001456
	SD	0.000019	0.000013	0.000019	0.000061	0.002533
	Min.	0.000144	0.000180	0.000184	0.000155	0.000159
	Max.	0.000264	0.000268	0.000327	0.000717	0.007328

SP	Ave.	0.707651	0.747853	0.711165	0.717493	0.752151
	SD	0.013739	0.015736	0.008840	0.032822	0.050459
	Min.	0.696859	0.715199	0.698590	0.697943	0.703181
	Max.	0.796404	0.793183	0.775317	0.950901	0.981492

HV	Ave.	0.512386	0.514813	0.513996	0.514996	0.493073
	SD	0.011314	0.000159	0.000675	0.001737	0.045211
	Min.	0.463776	0.514449	0.510764	0.500484	0.391275
	Max.	0.515960	0.515185	0.514668	0.515818	0.515941

**Table 10:** Performance comparison based on ZDT4 test problem.

Measure		AbYSS	NSGA-II	SPEA2	SMPSO	VEPSOml2
NS	Ave.	99.680000	100.000000	100.000000	100.000000	51.470000
	SD	3.100603	0.000000	0.000000	0.000000	35.623864
	Min.	69.000000	100.000000	100.000000	100.000000	4.000000
	Max.	100.000000	100.000000	100.000000	100.000000	100.000000

GD	Ave.	0.001231	0.000486	0.000923	0.0001347	0.449095
	SD	0.002632	0.000235	0.001428	0.000027	1.060986
	Min.	0.000148	0.000163	0.000176	0.000070	0.000146
	Max.	0.014472	0.001374	0.012292	0.000187	6.835452

SP	Ave.	0.159842	0.392885	0.298269	0.092281	0.735715
	SD	0.120180	0.037083	0.125809	0.011777	0.201246
	Min.	0.078244	0.324860	0.137934	0.067379	0.269484
	Max.	1.073669	0.473358	0.884091	0.124253	1.088971

HV	Ave.	0.646058	0.654655	0.645336	0.661401	0.307568
	SD	0.034449	0.003406	0.018773	0.000162	0.272435
	Min.	0.472299	0.642177	0.505799	0.660934	—
	Max.	0.661594	0.659710	0.658784	0.661726	0.660309

**Table 11:** Performance comparison based on ZDT6 test problem.

Measure		AbYSS	NSGA-II	SPEA2	SMPSO	VEPSOml2
NS	Ave.	100.000000	100.000000	100.000000	100.000000	88.590000
	SD	0.000000	0.000000	0.000000	0.000000	23.600674
	Min.	100.000000	100.000000	100.000000	100.000000	16.000000
	Max.	100.000000	100.000000	100.000000	100.000000	100.000000

GD	Ave.	0.000549	0.001034	0.001761	0.012853	0.217929
	SD	0.000015	0.000102	0.000192	0.024813	0.263966
	Min.	0.000510	0.000804	0.001267	0.000502	0.000482
	Max.	0.000596	0.001360	0.002207	0.092434	1.316312

SP	Ave.	0.097740	0.357160	0.226433	0.390481	1.273612
	SD	0.013129	0.031711	0.020658	0.497140	0.186562
	Min.	0.070455	0.282201	0.179482	0.042666	0.082405
	Max.	0.130389	0.441311	0.292897	1.377582	1.439400

HV	Ave.	0.400346	0.388304	0.378377	0.401280	0.315964
	SD	0.000172	0.001604	0.002714	0.000076	0.121842
	Min.	0.399821	0.383637	0.371907	0.401081	0.000001
	Max.	0.400842	0.392123	0.385626	0.401402	0.401483

## References

[1] Parsopóulos KE, Vrahatis MN Particle swarm optimization method in multiobjective problems.

[2] Gies D, Rahmat-Samii Y Vector evaluated particle swarm optimization (VEPSO): optimization of a radiometer array antenna.

[3] Rao SMV, Jagadeesh G (2010). Vector evaluated particle swarm optimization (VEPSO) of supersonic ejector for hydrogen fuel cells. *Journal of Fuel Cell Science and Technology*.

[4] Omkar SN, Mudigere D, Naik GN, Gopalakrishnan S (2008). Vector evaluated particle swarm optimization (VEPSO) for multi-objective design optimization of composite structures. *Computers & Structures*.

[5] Vlachogiannis JG, Lee KY (2009). Multi-objective based on parallel vector evaluated particle swarm optimization for optimal steady-state performance of power systems. *Expert Systems with Applications*.

[6] Grobler J (2009). *Particle swarm optimization and differential evolution for multi objective multiple machine scheduling [M.S. thesis]*.

[7] Ibrahim Z, Khalid NK, Mukred JAA (2012). A DNA sequence design for DNA computation based on binary vector evaluated particle swarm optimization. *International Journal of Unconventional Computing*.

[8] Ibrahim Z, Khalid NK, Buyamin S (2012). DNA sequence design for DNA computation based on binary particle swarm optimization. *International Journal of Innovative Computing, Information and Control*.

[9] Schaffer JD (1984). *Some experiments in machine learning using vector evaluated genetic algorithms (artificial intelligence, optimization, adaptation, pattern recognition) [Ph.D. thesis]*.

[10] Lim KS, Ibrahim Z, Buyamin S (2013). Improving vector evaluated particle swarm optimisation by incorporating nondominated solutions. *The Scientific World Journal*.

[11] Coello CAC, Lechuga MS Mopso: a proposal for multiple objective particle swarm optimization.

[12] Coello CAC, Pulido GT, Lechuga MS (2004). Handling multiple objectives with particle swarm optimization. *IEEE Transactions on Evolutionary Computation*.

[13] Li X, Cantú-Paz E, Foster J, Deb K (2003). A non-dominated sorting particle swarm optimizer for multiobjective optimization. *Genetic and Evolutionary Computation*.

[14] Deb K, Pratap A, Agarwal S, Meyarivan T (2002). A fast and elitist multiobjective genetic algorithm: NSGA-II. *IEEE Transactions on Evolutionary Computation*.

[15] Reyes-Sierra M, Coello CAC, Coello CAC, Aguirre AH, Zitzler E (2005). Improving PSO-based multiobjective optimization using crowding, mutation and *∈*-dominance. *Evolutionary Multi-Criterion Optimization*.

[16] Abido MA (2010). Multiobjective particle swarm optimization with nondominated local and global sets. *Natural Computing*.

[17] Kennedy J, Eberhart R Particle swarm optimization.

[18] Veldhuizen DAV (1999). *Multiobjective evolutionary algorithms: classifications, analyses, and new innovations [Ph.D. thesis]*.

[19] Zitzler E, Thiele L (1999). Multiobjective evolutionary algorithms: a comparative case study and the strength Pareto approach. *IEEE Transactions on Evolutionary Computation*.

[20] Zitzler E, Deb K, Thiele L (2000). Comparison of multiobjective evolutionary algorithms: empirical results. *Evolutionary Computation*.

[21] Zitzler E, Laumanns M, Thiele L, Giannakoglou KC (2002). Spea2: improving the strength pareto evolutionary algorithm for multiobjective optimization. *Evolutionary Methods for Design, Optimisation and Control with Application to Industrial Problems (EUROGEN '01)*.

[22] Nebro AJ, Luna F, Alba E, Dorronsoro B, Durillo JJ, Beham A (2008). AbYSS: adapting scatter search to multiobjective optimization. *IEEE Transactions on Evolutionary Computation*.

[23] Durillo J, García-Nieto J, Nebro A, Coello CAC, Luna F, Alba E, Ehrgott M, Fonseca C, Gandibleux X, Hao J-K, Sevaux M (2009). Multi-objective particle swarm optimizers: an experimental comparison. *Evolutionary Multi-Criterion Optimization*.

[24] Deb K (2001). *Multi-Objective Optimization Using Evolutionary Algorithms*.

[25] Nebro AJ, Durillo JJ, Nieto G, Coello CAC, Luna F, Alba E SMPSO: a new PSO-based metaheuristic for multi-objective optimization.

[26] Özcan E, Yılmaz M, Beliczynski B, Dzielinski A, Iwanowski M, Ribeiro B (2007). Particle swarms for multimodal optimization. *Adaptive and Natural Computing Algorithms*.

[27] Schoeman I, Engelbrecht A, Ribeiro B, Albrecht RF, Dobnikar A, Pearson DW, Steele NC (2005). A parallel vector-based particle swarm optimizer. *Adaptive and Natural Computing Algorithms*.

